# Short-term dietary choline supplementation alters the gut microbiota and liver metabolism of finishing pigs

**DOI:** 10.3389/fmicb.2023.1266042

**Published:** 2023-09-28

**Authors:** Zhongwei Xie, Junhua Du, Mailin Gan, Chengpeng Zhou, Menglin Li, Chengming Liu, Meng Wang, Lei Chen, Ye Zhao, Yan Wang, Yanzhi Jiang, Wenqiang Cheng, Kangping Zhu, Yi Luo, Li Zhu, Linyuan Shen

**Affiliations:** ^1^Key Laboratory of Livestock and Poultry Multi-omics, Ministry of Agriculture and Rural Affairs, College of Animal and Technology, Sichuan Agricultural University, Chengdu, China; ^2^State Key Laboratory of Swine and Poultry Breeding Industry, Sichuan Agricultural University, Chengdu, China; ^3^College of Life Science, Sichuan Agricultural University, Chengdu, China; ^4^National Animal Husbandry Service, Beijing, China; ^5^Sichuan Dekon Livestock Foodstuff Group, Shuangliu, China

**Keywords:** choline, body weight, gut microbiota, liver metabolism, finishing pigs

## Abstract

Choline is an essential nutrient for pig development and plays a role in the animal's growth performance, carcass characteristics, and reproduction aspects in weaned pigs and sows. However, the effect of choline on finishing pigs and its potential regulatory mechanism remains unclear. Here, we feed finishing pigs with 1% of the hydrochloride salt of choline, such as choline chloride (CHC), under a basic diet condition for a short period of time (14 days). A 14-day supplementation of CHC significantly increased final weight and carcass weight while having no effect on carcass length, average backfat, or eye muscle area compared with control pigs. Mechanically, CHC resulted in a significant alteration of gut microbiota composition in finishing pigs and a remarkably increased relative abundance of bacteria contributing to growth performance and health, including Prevotella, Ruminococcaceae, and Eubacterium. In addition, untargeted metabolomics analysis identified 84 differently abundant metabolites in the liver between CHC pigs and control pigs, of which most metabolites were mainly enriched in signaling pathways related to the improvement of growth, development, and health. Notably, there was no significant difference in the ability of oxidative stress resistance between the two groups, although increased bacteria and metabolites keeping balance in reactive oxygen species showed in finishing pigs after CHC supplementation. Taken together, our results suggest that a short-term supplementation of CHC contributes to increased body weight gain and carcass weight of finishing pigs, which may be involved in the regulation of gut microbiota and alterations of liver metabolism, providing new insights into the potential of choline-mediated gut microbiota/metabolites in improving growth performance, carcass characteristics, and health.

## 1. Introduction

Choline, with a molecular formula of (CH_3_)_3_N(CH_2_)_2_OH, is a water-soluble vitamin that plays a vital role in maintaining physiological functions in organisms, including humans and livestock (Chin et al., 2019). While animals can typically synthesize choline on their own, there are certain physiological conditions, such as energy imbalance, where supplementing additional choline in the diet becomes necessary to ensure the normal physiological function of animals (Bryant et al., 1999; Zenobi et al., 2018). In animal production, choline chloride (CHC), which is the hydrochloride salt of choline, is frequently employed as a dietary additive (Yara et al., 2015). It was typically added separately to avoid any potential adverse impacts on other vitamins. The liver serves as the primary site for the oxidation and phosphorylation metabolism of choline (Mehedint and Zeisel, 2013). In cases of inadequate dietary choline, the liver employs phosphatidylethanolamine N-methyltransferase to facilitate the transfer of a methyl group, resulting in the formation of phosphatidylcholine. Phosphatidylcholine can serve as a precursor for the synthesis of very low-density lipoprotein, enabling the transportation of triglycerides to prevent the excessive buildup of triglycerides and the onset of fatty liver (Li et al., 2005; Vance, 2008). Alternatively, through the action of phospholipase D, it can be converted back to choline for utilization by various tissues in the body (Ridgway, 2013). Therefore, choline plays a role in promoting fat transport (Chen et al., 2021) and enhancing liver fat metabolism (Corbin and Zeisel, 2012). In livestock and poultry research, choline has also been shown to improve production performance (Li et al., 2018), maintain cell membrane integrity (Corbin and Zeisel, 2012), and provide resistance against various stresses (Zheng et al., 2016; Hussain et al., 2021). Research indicated that pigs have substantial agricultural importance globally and are increasingly employed as models for human diseases (Groenen et al., 2012). However, the current research findings regarding the impact of choline on growth performance and lipid metabolism in piglets show inconsistencies (Siljander-Rasi et al., 2003). In pig farming, finishing pigs play a crucial role in the final stage, and their production performance has a substantial impact on the economic profitability of a pig farm. However, the research on the effects of choline on the growth performance of finishing pigs is currently limited. It is not clear whether choline will have inconsistent results in fattening pig research due to the feeding cycle and feeding measurement. Furthermore, further investigation is needed to elucidate the underlying mechanisms through which choline regulates growth performance in the context of finishing pig research.

The production performance of pigs relies on the efficient digestion and absorption of nutrients within their bodies (Cheng et al., 2021; Ma et al., 2022). During the process of nutrient digestion and absorption, the microorganisms present in the intestinal tract of pigs play various roles. Under normal conditions, the microorganisms residing in the intestinal tract maintain a stable and mutually beneficial symbiotic relationship with the host. However, any disruption to this balance can result in host dysfunction and give rise to intestinal diseases (Wang et al., 2017). Multiple studies have indicated that a healthy microbiome in the intestinal tract of finishing pigs has a beneficial impact on weight gain, feed efficiency, and feed intake. Among the factors influencing intestinal microbes, diet plays a significant role in modulating the composition, diversity, and functionality of the intestinal microbial communities. Consequently, this leads to enhanced intestinal health and the maintenance of energy homeostasis. Zhan et al. (2023) observed an increase in the diversity of intestinal microbiota after feeding gestating sows with 500 mg/kg of choline chloride for 96 days. Qiu et al. (2021) found that feeding weaned piglets with 597 mg/kg of choline chloride for 28 days resulted in a decrease in the richness and diversity indices of the intestinal microbiota compared to the feed without choline supplementation. These findings suggest that the influence of choline on the pig's intestinal microbiota may be dependent on factors such as the pig's growth stage, feeding duration, and dosage (Qiu et al., 2021; Zhan et al., 2023). However, there is still limited research on the effects of choline on the intestinal microbiota of finishing pigs.

In this study, we primarily focused on finishing pigs and conducted a 14-day supplementation trial with choline. The aim was to investigate the effects of choline supplementation on the growth performance of finishing pigs, changes in the intestinal microbiota, and alterations in liver metabolites. This research has conferred valuable insights into the impact of choline supplements on pigs at different growth stages, thereby enhancing our knowledge and understanding of the role of choline in swine nutrition.

## 2. Materials and methods

### 2.1. Animals and experimental design

After male large white pigs (~160 days of age) from the same farrowing batch were adaptively reared for 11 days, a total of 20 pigs without significant difference in body weight were assigned to two dietary groups (*n* = 10 per group), including a basal diet or a basal diet + 1% choline chloride, as outlined by Yoo et al. (2021). The procedure involved blending water and dry components with liquid feed in a ratio of 3:1. Subsequently, 10 g of choline chloride was incorporated with 1 kg of the liquid feed. The composition and nutrient levels of the basal diet was supplemented by Supplementary Table 1. Each group of 10 pigs was divided and housed in two separate pens. All pigs were provided with *ad libitum* access to feed and water and were housed under the same environmental conditions of 25 ± 2°C. The live body weight of the finishing pigs was measured using the pig individual weighing device D600 (Jiangsu Kono Animal Husbandry Equipment Technology Co., Ltd., China) in the morning. Choline chloride, characterized by a minimum purity of 98%, was sourced from Shanghai Yuanye Biological Co., Ltd. (Shanghai, China). All pigs from the two diet groups were fed with a basal diet or a basal diet + 1% choline chloride for 14 days.

All pigs were provided by a pig breeding company in Sichuan Province, China. All the experimental procedures were approved by the Animal Ethical and Welfare Committee of Sichuan Agricultural University, Chengdu, China (approval number 2021302172).

### 2.2. Animal analysis and sample collection

Fresh feces collected from finishing pigs were immediately frozen and stored at −80°C for DNA extraction. Subsequently, the carcass characteristics were evaluated. Briefly, all pigs were moved to a commercial slaughter room (Mianyang City, Sichuan province, China), electrically stunned, and then slaughtered according to standard commercial procedures. The live body weight and hot carcass weight were immediately recorded for the dressing percentage calculation. Meanwhile, carcass length was also measured. Backfat thickness and eye muscle area were quantified by employing a vernier caliper (Guilin Guanglu Measuring Instrument Co., Ltd., Guilin, China). Backfat thickness (mm) was calculated by averaging the scores of three regions at the first rib, last rib, and last lumbar vertebrae of the right carcass sides (Li et al., 2018). The eye muscle area was measured at the last rib using vernier calipers. In addition, we collected fresh livers from finishing pigs and rapidly froze them using liquid nitrogen before storing them at −80°C for further analysis of antioxidant indicators and metabolites.

### 2.3. Analysis of antioxidant parameters (TG and TCHO)

The liver samples were mixed with nine times the volume of normal saline and prepared as a 10% tissue homogenate. All procedures were carried out in an ice water bath. Following centrifugation at 2,500 *g* for 10 min, the resulting supernatant was collected in a fresh centrifuge tube and stored at 20°C for subsequent analysis of antioxidant parameters. Then, superoxide dismutase (SOD), glutathione peroxidase (GSH-Px), malonaldehyde (MDA), total antioxidant capacity (T-AOC), triglyceride (TG), and total cholesterol (TCHO) were examined using the commercial kits (Nanjing Jiancheng Co., Ltd., Nanjing, China), according to the manufacturer's instructions. Moreover, the protein content of the liver samples was assessed using a BCA protein assay kit (Biosharp, Beijing, China). The GSH-Px, SOD, and T-AOC levels were expressed as units/mg protein. The TG and TCHO levels were expressed as mmol/g protein. The MDA level was expressed as nmol/mg protein.

### 2.4. Gut microbial profiling

In brief, after extraction of the total genome DNA from feces using the CTAB method (Raimundo et al., 2018), DNA concentration and purity were monitored on 1% agarose gels. According to the concentration, DNA was diluted to 1 ng/μl with sterile water. Subsequently, 16S rRNA genes in distinct regions (16S V3–V4) were amplified with specific primers (341F-806R) and barcodes. The PCR products were mixed in equal proportions and purified using the Qiagen Gel Extraction Kit (Qiagen, Hilden, Germany).

The sequencing library was sequenced on an Illumina NovaSeq platform, and 250-bp paired-end reads were generated after libraries were generated with the NEBNext^®^Ultra™ IIDNA Library Prep Kit (Cat No. E7645) following the manufacturer's recommendations. For the effective tags obtained previously, denoise was performed with DADA2 or the deblur module in the QIIME2 software (version QIIME2-202006) to obtain initial Amplicon Sequence Variants (ASVs; default: DADA2), and then ASVs with an abundance less than five were filtered out (Li M. et al., 2020). To assess the phylogenetic relationship of each ASV and the differences between the dominant species among different samples, multiple sequence alignment was performed using the QIIME2 software. Alpha diversity (within sample) analysis, including Good's coverage, Chao1, and Shannon, was calculated using QIIME2. To evaluate the complexity of the community composition and compare the differences between samples (groups), principal coordinate analysis (PCoA) and non-metric multidimensional scaling (NMDS) were calculated based on unweighted UniFrac distances in QIIME2. To find out the significantly different species at each taxonomic level (Phylum, Family, and Genus), the R software (version 3.5.3) was performed using MetaStat and *T*-test analysis, as well as the linear discriminant analysis (LDA) effect size software (version 1.0). The microbial biomarkers were discovered using the LEfSe analysis (LDA score threshold: 4). The prediction of metagenome functional content from the 16S rRNA library was developed using the Phylogenetic Investigation of Communities by Reconstruction of Unobserved States (PICRUSt) software (Version 2.1.2-b), and PICRUSt predictions were categorized as levels 2 into Kyoto Encyclopedia of Genes and Genomes (KEGG) pathways.

### 2.5. Metabolomics analyses

#### 2.5.1. Extraction of metabolites

The metabolites were extracted from the liver with 1 ml precooled methanol/acetonitrile/water (v/v, 2:2:1) under sonication for 1 h in ice baths. The mixture was incubated at −20°C for 1 h followed by centrifugation at 16,000 *g* for 20 min at 4°C and then transferred to the sampling vial for LC-MS analysis. Quality control (QC) samples were prepared by pooling aliquots of all samples that were representative of all the samples under analysis and used for data normalization.

#### 2.5.2. UHPLC-MS analysis

Untargeted metabolomics measurement was performed by ultra-high-performance liquid chromatography/time-of-flight mass spectrometry (UHPLC-MS). Briefly, the LC/MS portion of the platform was based on a Shimadzu Nexera X2 LC-30AD system equipped with an Acquity UPLC HSS T3 column (1.8 μm 2.1 × 50 mm Column, Waters) and a triple quadruple mass spectrometer (5500 QTRAP, AB SCIEX). Metabolites were detected in electrospray negative-ionization and positive-ionization modes. The 5 μl samples were injected sequentially with an LC autosampler. The Acquity UPLC HSS T3 column (1.8 μm 2.1 × 50 mm Column, Waters) was heated to 40°C under a flow rate of 200 μl/min. A gradient was used to separate the compounds, which consisted of 0.1% formic acid aqueous solution (solvent A) and 100% acetonitrile (solvent B). The gradient started at 100% solvent A for 2.5 min, increasing linearly to 70% solvent A over 9 min, and then increasing linearly to 0% solvent A over 1 min, following a 5.4-min hold before returning the starting mixture during 0.1 min and re-equilibrating for 2.5 min. QC samples were injected into several samples during acquisition.

The MS conditions were set as follows: negative-ionization: source temperature, 550°C; ion source gas1 (GAS1), 40; ion source gas2 (GAS2), 50; curtain gas (CUR), 35; and ion spray voltage floating (ISVF), −4,500 V; and positive-ionization: source temperature, 550°C; ion source gas1 (GAS1), 40; ion source gas2 (GAS2), 50; curtain gas (CUR), 35; and ion spray voltage floating (ISVF), 5,500 V. Transitions were detected by MRM mode.

#### 2.5.3. Data preprocessing and filtering

The MultiQuant 3.0.2 software was used to extract the original MRM data of MT1000 KIT metabolites and obtain the peak area of each metabolite. Next, the discriminating metabolites were obtained using a statistically significant threshold of variable influence on projection (VIP) values obtained from the OPLS-DA model and a two-tailed Student's *t*-test (*p*-value) on the normalized raw data. The *p*-value was calculated using a one-way analysis of variance (ANOVA) for multiple-group analysis. Metabolites with a VIP >1 and a *p*-value of < 0.05 were considered to be statistically significant metabolites. The identified differential metabolites were used to perform cluster analyses with the R package. Finally, the differential metabolite data were subjected to KEGG pathway analysis using the KEGG database (http://kegg.jp). KEGG enrichment analyses were carried out with Fisher's exact test, and FDR correction for multiple testing was performed. Enriched KEGG pathways were nominally statistically significant at the *p* < 0.05 level.

### 2.6. Statistical analyses

Data are presented as the means ± SD. The data between the two groups were analyzed using a two-tailed Student's *t*-test using SPSS 20.0 (SPSS, Chicago, IL, United States). *p*-values of < 0.05 were considered to be significant. Pearson correlation analysis was employed to analyze the correlation between intestinal microorganisms and liver metabolites.

## 3. Results

### 3.1. Dietary CHC increases the body weight of finishing pigs

To investigate the effect of CHC on finishing pigs, finishing pigs born on the same day were assigned to a basal diet (NC) or NC containing 1% choline chloride (CHC) for a short term of 14 days, respectively. As shown in Table 1, finishing pigs fed with CHC exhibited significantly increased final weight when compared to NC pigs. To gain more insights into the impact of CHC on the body weight of finishing pigs, we evaluated carcass characteristics and found that CHC resulted in a marked increase in carcass weight but not in dressing percentage when compared to finishing pigs fed with NC. Likewise, finishing pigs fed with CHC had a relatively higher carcass length and lower average backfat than that of NC pigs. However, no difference was detected in the eye muscle area between the two diet groups.

**Table 1:** Effect of CHC on the body weight of finishing pigs.

**Item**	**Group**		***p*** **-value**
	**NC (*****n*** = **10)**	**CHC (*****n*** = **10)**	
Final weight, kg	106.80 ± 8.02	115.80 ± 7.77	<0.05
Carcass weight, kg	78.12 ± 6.86	85.85 ± 5.85	<0.05
Dressing percentage, %	73.10 ± 0.02	74.17 ± 0.03	0.29
Carcass length, cm	99.90 ± 5.24	104.10 ± 4.43	0.07
Average backfat, cm	1.84 ± 0.23	1.66 ± 0.27	0.12
Eye muscle area, cm^2^	33.04 ± 13.72	33.18 ± 10.90	0.98

### 3.2. Alterations of the gut microbiota of finishing pigs upon dietary CHC

**Figure 1:**
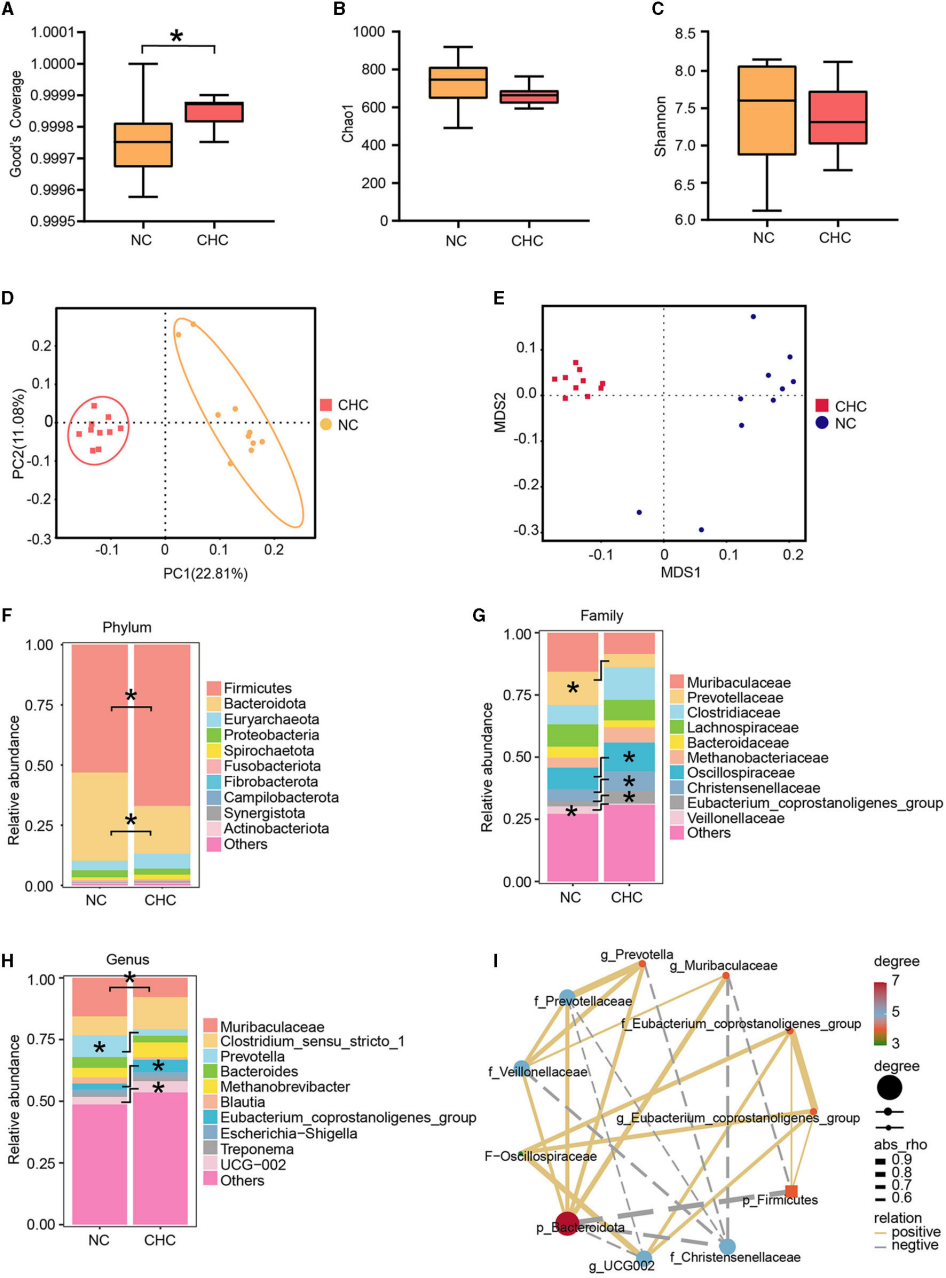
Effects of CHC supplementation on the composition of the gut microbiota. **(A–C)** Alpha diversity analysis of Good's coverage, Chao1, and Shannon index. **(D)** Principal coordinates analysis (PCoA). **(E)** Non-metric multidimensional scaling (NMDS). **(F–H)** Shifts in the relative abundance of bacterial taxa. The microbiota composition at the phylum **(F)**, family **(G)**, and genus **(H)** levels. **(I)** Pearson correlation analysis of two significant phyla, five significant families, and four significant genera. *p < 0.05.

Given growing evidence showing a critical role of gut microbiota in the growth, development, and health of humans and animals, especially CHC, which regulates host metabolism by interacting with gut microbiota, 16S rRNA profiling was performed on DNA extracted from fecal samples to discern and characterize the gut microbiota between NC pigs and CHC pigs. To this end, we evaluated alpha diversity through specific indices such as sequencing depth (Good's coverage), richness (Chao1), and evenness (Shannon). As shown in Figures 1A–C, Good's coverage revealed sufficient sequencing depth in all samples, while there were no significant changes in Chao1 and Shannon indexes between the two diet groups, suggesting that dietary CHC supplementation may not affect the diversity and richness of the gut microbiota in finishing pigs. Subsequently, we analyzed the overall changes in the microbial community of finishing pigs exposed to dietary CHC by performing both principal coordinate analysis (PCoA) and non-metric multidimensional scaling (NMDS). The PCoA results revealed a significant alteration of microbiota composition in finishing pigs after CHC exposure (Figure 1D), as confirmed by NMDS (Figure 1E). Notably, in accordance with the shift observed in the PCoA result, exposure to CHC resulted in altering the relative abundance of bacteria at different taxonomic levels.

At the phylum level, the gut microbiota of finishing pigs exposed to both NC and CHC were dominated by the phyla of Bacteroidota and Firmicutes, with a lower proportion of Bacteroidota and a higher relative abundance of Firmicutes in CHC pigs compared to NC pigs (Figure 1F). Given that the significantly higher abundance ratio of Firmicutes to Bacteroidota is generally regarded as a marker signal of metabolic health, we conducted a thorough investigation of the F:B ratio in both groups. The F:B ratio clearly increased from 1.86 in the NC group to 3.83 in the CHC group (Supplementary Figure 1A), suggesting a major contribution of CHC to improve the metabolism of finishing pigs. Among the top 40 abundant genera contained in Firmicutes and Bacteroidetes, a total of 11 genera were altered by CHC supplementation (Supplementary Figures 1B-L). At the family level, exposure to CHC contributed to an increase in Oscillospiraceae, Christensenellaceae, and Eubacterium coprostanoligenes group, but also caused a marked reduction in the relative abundance of Veillonellaceae, especially Prevotellaceae in finishing pigs (Figure 1G). Interestingly, further analysis at the genus level showed that four genera of bacteria were significantly different among the groups. The relative abundance of Muribaculaceae (from 15.59 to 7.76%) and Prevotella (from 8.77 to 2.68%) significantly decreased in the CHC pigs compared with those in the control group, whereas the relative abundance of Eubacterium coprostanoligenes group and UCG-002 was significantly increased (Figure 1H).

To better understand the effect of CHC on gut microbiota in finishing pigs, we next investigated the interaction between bacteria responding to CHC. To address this, Pearson correlation analysis was further performed to examine the associations among significant phyla, families, and genera between two diet groups. As shown in Figure 1I, p_Bacteroidota showed a positive correlation with f_Veillonellaceae, f_Prevotellaceae, g_Prevotella, and g_Muribaculaceae, while it was negatively correlated with p_Firmicutes, f_Christensenellaceae, and g_UCG002. In addition, f_Oscillospiraceae exhibited a positive correlation with f_Eubacterium coprostanoligenes group, g_Eubacterium coprostanoligenes group, and g_UCG002.

### 3.3. Identification of gut microbiome biomarkers and functional alterations of gut microbiota in CHC pigs

**Figure 2:**
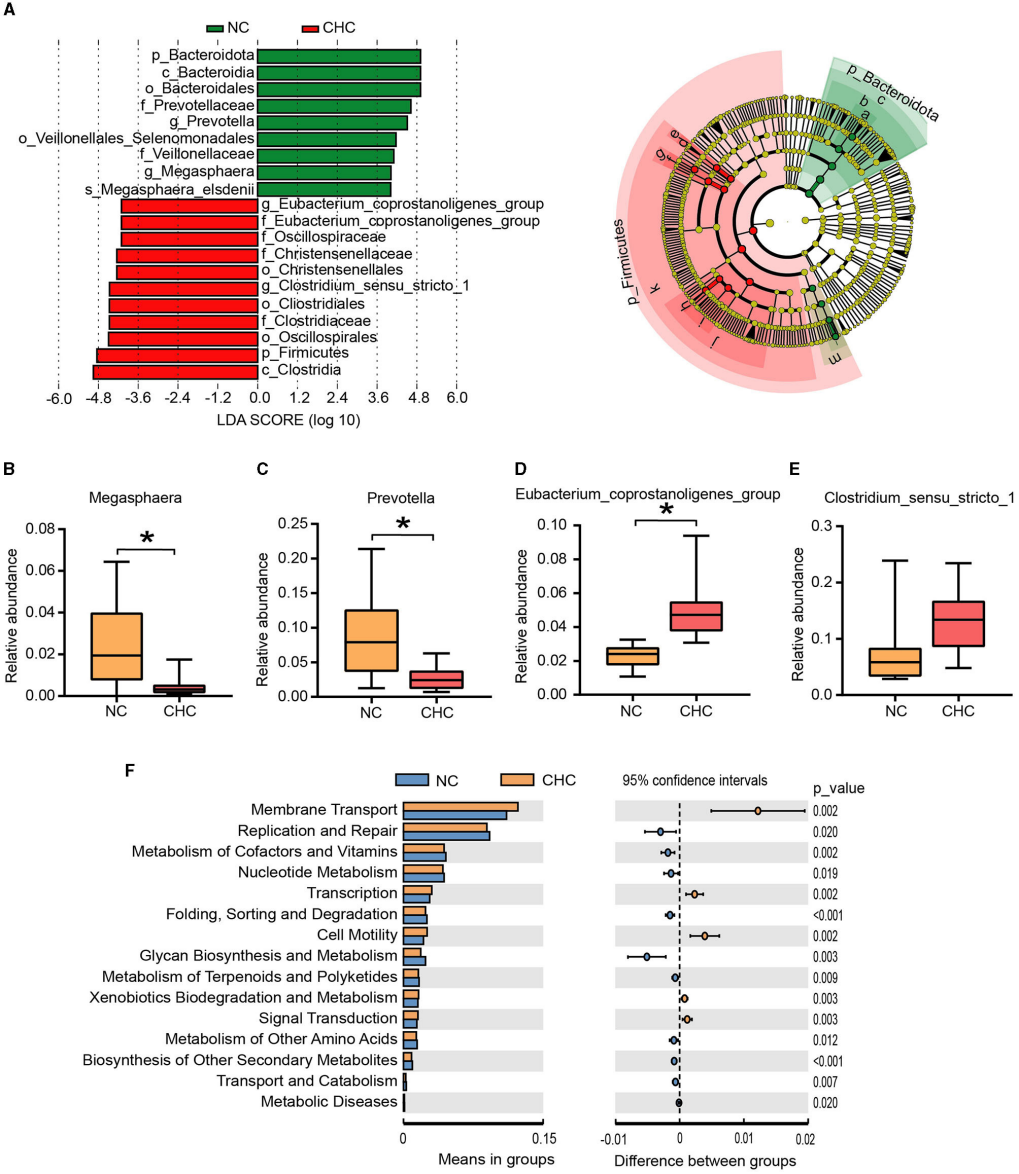
Identification of gut microbiome biomarkers and functional prediction analysis using PICRUSt2. **(A)** Linear discriminant analysis effect size (LEfSe) analysis of key bacteria of the gut microbiota in fattening pigs and the LDA score >4. **(B–E)** Significant changes (*p* < 0.05) in the relative abundance of four potential genera. **(F)** PICRUSt2 function prediction of the 16S rDNA gene from the gut microbiota. *p < 0.05.

We used the linear discriminant analysis effect size (LEfSe) algorithm to perform LDA to identify operational microbiotas that are differentially abundant with regard to CHC treatment, based on the significantly different species screened at different taxonomic levels. A total of 20 potential biomarkers were observed between the two groups (Figure 2A). Consistent with the observation in Figure 1I, p_Bacteroidota, c_Bacteroidia, o_Bacteroidales, f_Prevotellaceae, g_Prevotella, o_Veillonellales Selenomonadales, f_Veillonellaceae, g_Megasphaera, and s_Megasphaera elsdenii were enriched in the NC pigs, while significantly higher, g_Eubacterium coprostanoligenes group, f_Eubacterium coprostanoligenes group, f_Oscillospiraceae, f_Christensenellaceae, o_Christensenellales, g_Clostridium sensu stricto_1, o_Cliostridiales, f_Clostridiaceae, o_Oscillospirales, p_Firmicute, and c_Clostridia exhibited in the CHC pigs. At the genus level, a lower relative abundance of Prevotella and Megasphaera and a higher relative abundance of Eubacterium coprostanoligenes group were shown in fecal samples of CHC pigs compared to NC pigs (Figures 2B–E).

Subsequently, the PICRUST analysis was employed to clarify functional alterations in the gut microbiota responding to CHC exposure. As shown in Figure 2F, a *t*-test analysis was conducted on the abundance of annotated KEGG levels (level 2) and showed that gut microbiotas enriched in fecal samples of CHC pigs were involved in five metabolic pathways, including membrane transport, transcription, cell motility, xenobiotic biodegradation, metabolism, and signal transduction. In contrast, the top discriminative microbial pathways in NC pigs included pathways mainly related to replication and repair, and biosynthesis and metabolism of glycan.

### 3.4. Altered metabolic profiles of the liver in finishing pigs upon CHC

**Figure 3:**
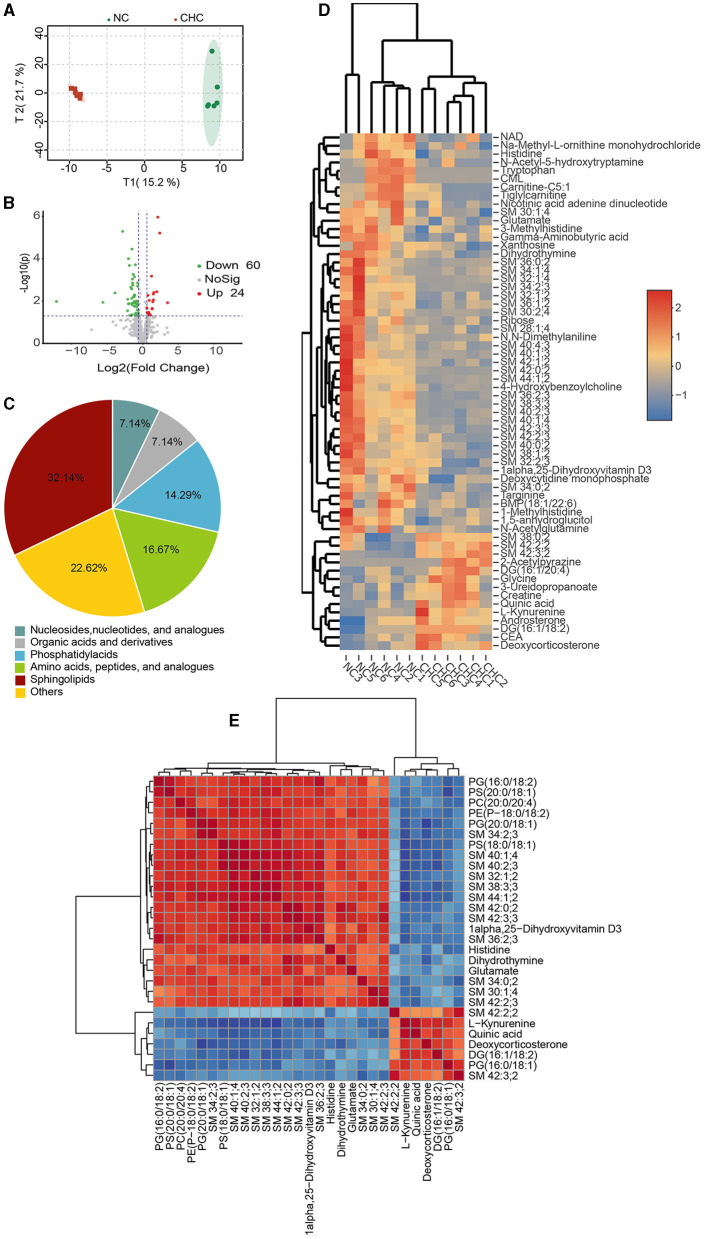
Liver metabolic traits in response to CHC in finishing pigs. **(A)** OPLS-DA analysis of identified metabolites. **(B)** Volcano map of metabolites. **(C)** Number of identified metabolites in liver samples and distributions of chemical classes. **(D)** Heatmap analysis was conducted to visualize the differences in the abundance of 84 metabolites following CHC treatment. **(E)** Pearson correlation analysis was performed to assess the correlation between the identified top 30 metabolites based on VIP values.

It is well-known that the liver, a major metabolic site of the whole body, can produce choline, which also plays an important role in regulating several vital biological functions of the liver. To verify whether CHC-mediated body weight gain was involved in the alteration of liver metabolism, we performed untargeted metabolomics analysis on hepatic tissues from NC pigs and CHC pigs to identify differences in metabolites. A total of 594 metabolites were detected following the guidelines of the Metabolomics Standards Initiative (MSI), and the OPLS analysis exhibited a clear separation of metabolic profiles between the two diet groups (Figure 3A). Using VIP scores derived from the OPLS-DA model with a VIP value of ≥1.0 and a *p*-value of < 0.05, we next identified metabolites associated with the CHC treatment. As shown in Figures 3B, C, 84 significantly and differently abundant metabolites were found in the liver of NC pigs and CHC pigs, which belonged predominantly to three major nutrient classes, namely, sphingolipids (32, 14%), amino acids, peptides, and analogs (16.67%), as well as phosphatidyl acids (14.29%). Among altered metabolites responding to CHC exposure, 24 metabolites such as deoxycorticosterone, CEA, and androsterone significantly increased and 60 metabolites, including NAD, remarkably decreased in the liver of CHC pigs when compared to that of NC pigs (Figure 3D). It was important to note that the correlation analysis of the top 30 metabolites altered by CHC clearly revealed a strong and positive correlation between sphingolipid metabolites (SM 34:0;2, SM 36:2;3, SM 38:3;3, SM 42:2;2, SM 40:2;3, SM 42:3;3, SM 42:2;3, and SM 34:2;3) and phosphatidyl acid metabolites [PS (20:0/18:1), PG (16:0/18:2), PE (P-18:0/18:2), PS (18:0/18:1), and PG (20:0/18:1); Figure 3E]. Interestingly, there was also a positive correlation among the sphingolipid metabolites themselves (SM 34:0;2, SM 36:2;3, SM 38:3;3, SM 42:2;2, SM 40:2;3, SM 42:3;3, SM 42:2;3, and SM 34:2;3; Figure 3E). In relative to a positive correlation between DG (16:1/18:2), SM 42:3;2, and PG (16:0/18:1), these three metabolites negatively correlated with sphingolipid metabolites (SM 34:0;2, SM 36:2;3, SM38:3;3, SM 42:2;2, SM 40:2;3, SM 42:3;3, SM 42:2;3, and SM 34:2;3) and phosphatidyl acids metabolites [PS (20:0/18:1), PG (16:0/18:2), PE (18:0/18:2), PS (18:0/18:1), and PG (20:0/18:1); Figure 3E].

**Figure 4:**
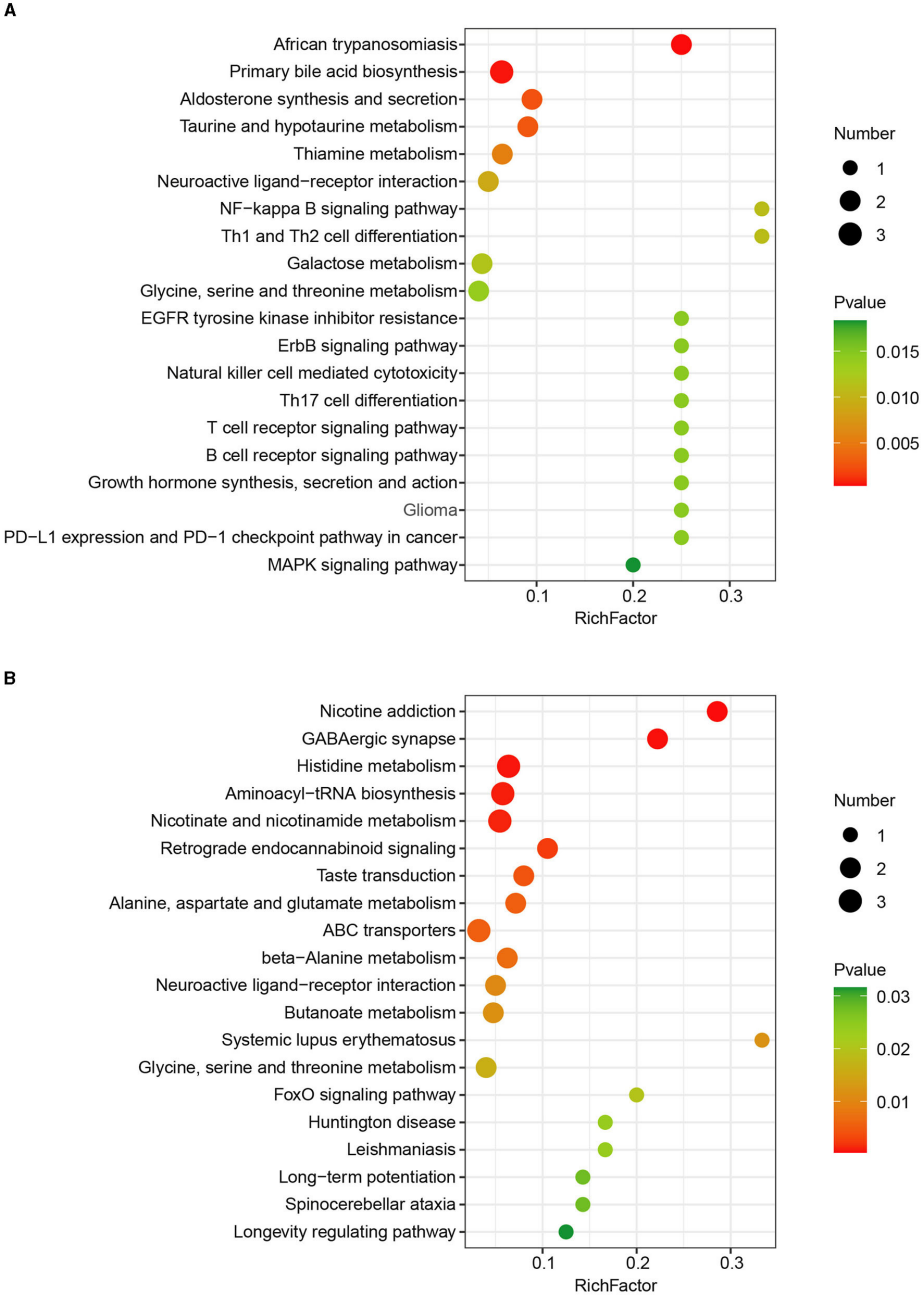
KEGG pathway enrichment analysis of differentially expressed metabolites. **(A)** KEGG pathway enrichment analysis of upregulated metabolites. **(B)** KEGG pathway enrichment analysis of downregulated metabolites.

To better understand the determined metabolites significantly associated with CHC in the liver of finishing pigs, metabolite set enrichment analysis of the differentially abundant metabolites between the two groups was further performed based on the KEGG pathways. In total, we detected 40 significantly altered metabolic KEGG pathways in the liver in response to CHC exposure, in which significantly increased metabolites were mainly involved in African trypanosomiasis, primary bile acid biosynthesis, aldosterone synthesis and secretion, taurine and hypotaurine metabolism, and thiamine metabolism (Figure 4A). In contrast, reduced metabolites by CHC exposure significantly mediated nicotine addiction, GABAergic synapse, histidine metabolism, aminoacyl-tRNA biosynthesis, and nicotinate and nicotinamide metabolism (Figure 4B). Taken together, these data indicate that CHC treatment can significantly alter liver metabolism in finishing pigs.

### 3.5. CHC treatment promotes taurine metabolism in the liver of finishing pigs

**Figure 5:**
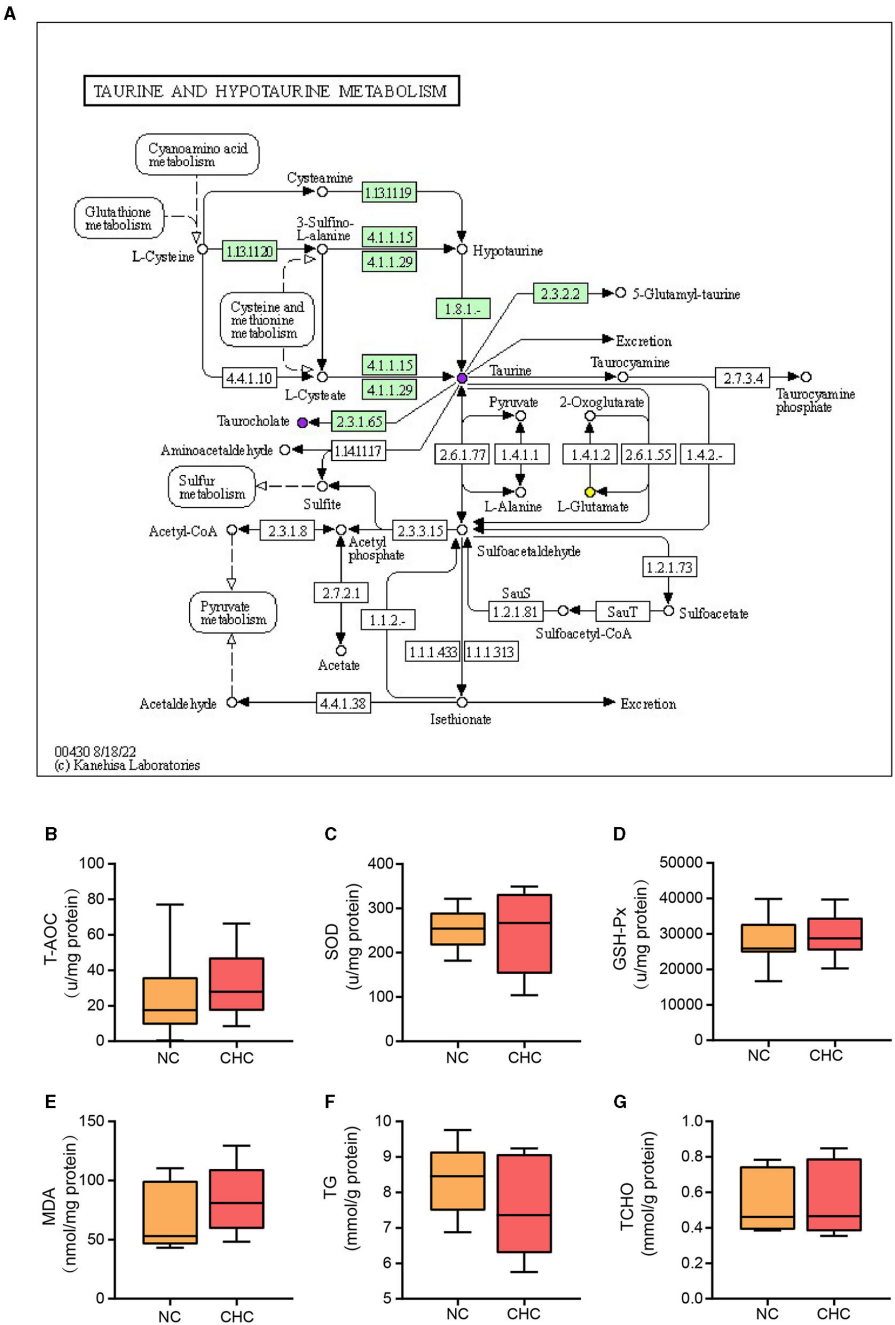
CHC treatment promotes taurine metabolism in the liver of finishing pigs. **(A)** The differential metabolites were annotated to the taurine and hypotaurine metabolism pathways. The upregulated significantly different metabolites are represented by purple dots, while the downregulated significantly different metabolites are represented by yellow dots. Effects of CHC supplementation on liver oxidative stress **(B–E)** and TG and TCHO contents in the liver **(F, G)**.

Taurine, a semi-essential micronutrient, can be converted into hypotaurine through methionine in the liver (Stipanuk et al., 2006). Among the significantly enriched signaling pathways involved in hepatic metabolites altered by CHC, we found that taurine and hypotaurine metabolism were involved in cyanoamino acid metabolism, glutathione metabolism, cysteine and methionine metabolism, sulfur metabolism, and pyruvate metabolism. In particular, increased choline chloride may contribute to the upregulation of taurine by regulating cyanoamino acid metabolism and glutathione metabolism (Figure 5A). It is well-known that excessive production of reactive oxygen species (ROS) in the liver, coupled with a compromised antioxidant mechanism, can lead to oxidative stress, which adversely affects liver synthesis and metabolism, resulting in a decline in the growth performance of pigs. Furthermore, the liver, being a crucial site for lipid metabolism, is susceptible to lipid metabolism disorders during oxidative stress, which can also have a detrimental impact on the growth performance of pigs. Given the importance of taurine and hypotaurine metabolism in the regulation of ROS-driven liver damage, we, therefore, determined whether CHC can affect the antioxidant capacity and lipid metabolism of the liver in finishing pigs during growth. To this end, we compared total antioxidant capacity (T-AOC), glutathione peroxidase (GSH-PX), malondialdehyde (MDA), superoxide dismutase (SOD), triglyceride (TG), and total cholesterol (TCHO) in the liver between two groups. As shown in Figures 5B–G, no significant difference was found in all indicators examined between the two groups. However, there was an increasing trend in T-AOC, GSH-PX, and SOD levels but a decreasing trend in TG levels, suggesting that CHC may have a potential effect in reducing oxidative stress and promoting body weight gain in finishing pigs.

### 3.6. Correlations between gut microbiota and metabolites

**Figure 6:**
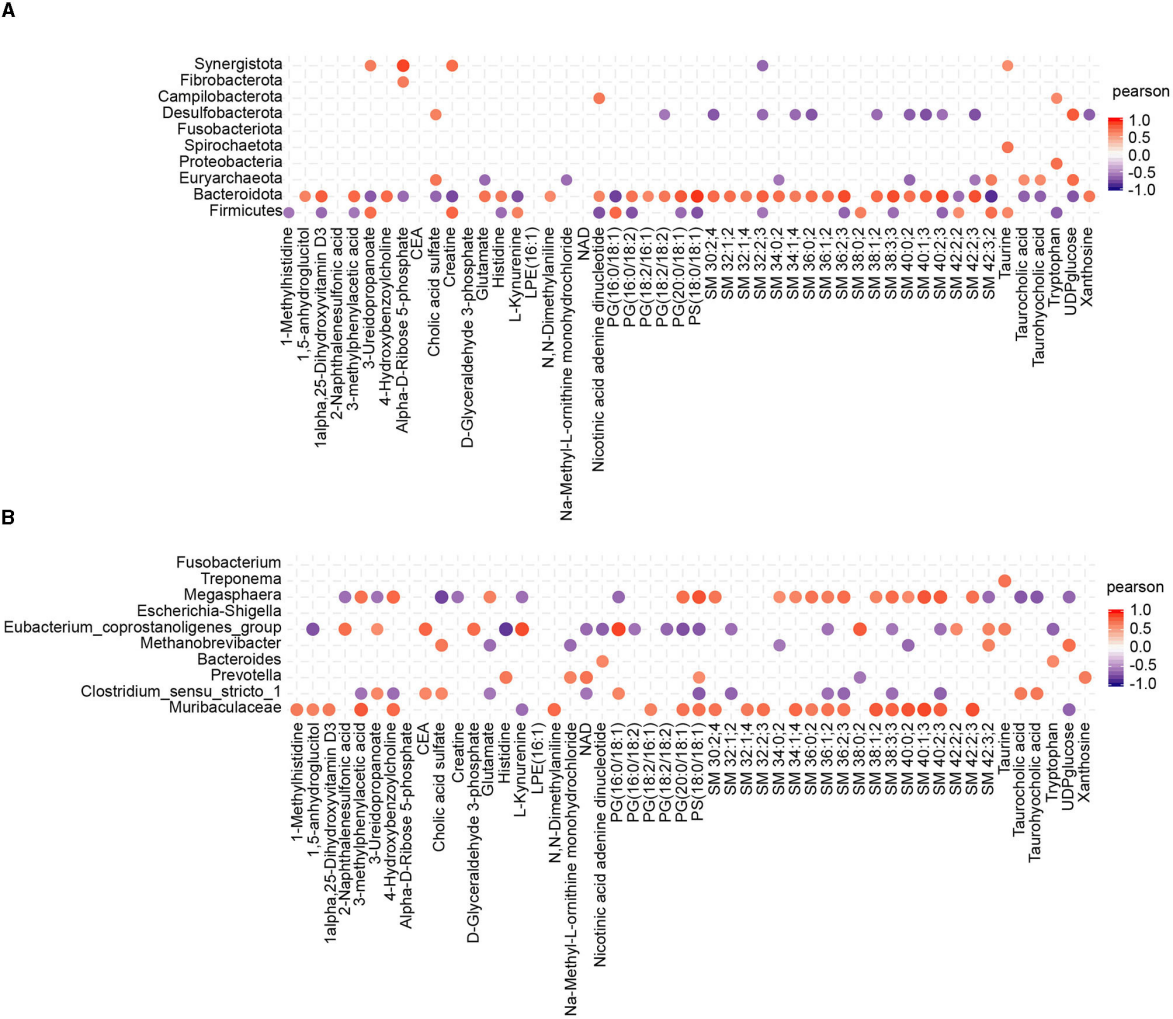
Statistical Pearson correlation analysis between the gut microbiota and differential metabolites. **(A)** Correlation analysis between the top 10 phyla and metabolites. **(B)** Correlation analysis between the top 10 genera and metabolites.

To investigate the potential relationship between the alterations in gut microbiota induced by CHC and the resulting effects on metabolites, Pearson correlation analysis was performed to examine the correlations between the composition of gut microbiota (at the phylum and genus levels) and the differential metabolites. In the generated heatmap matrix, the color red represents positive correlations between the bacterial taxa and metabolites, while the color blue indicates negative correlations. At the phylum level, Bacteroidota was strongly positively correlated with sphingolipid and phosphatidyl acid metabolites, while Desulfobacterota was negatively correlated with sphingolipid metabolites (Figure 6A). At the genus level, Muribaculaceae and Megasphaera were functionally similar and strongly positively correlated with sphingolipid metabolites. Furthermore, Muribaculaceae showed a positive correlation with 1-methylhistidine, 1,5-anhydroglucitol, 1alpha,25-dihydroxyvitamin D3, 3-methylphenylacetic acid, and 4-hydroxybenzoylcholine (Figure 6B).

## 4. Discussion

Growth performance and carcass characteristics are crucial factors for assessing the efficiency of livestock and poultry production as well as meat quality. Previously, studies have revealed a significant association between choline intake and body weight (Igwe et al., 2015; Bagley et al., 2017). Jiao et al. (2018) reported that a 56-day dietary supplementation of 0.05% CHC alone resulted in an increased average daily gain for finishing pigs. The same is also confirmed by a recent study, in which 0.05% CHC supplementation for 96 days can significantly increase overall body weight gain (Zhan et al., 2023). However, some recent studies showed the opposite effect of CHC on the body weight of piglets, which may be due to variations in the composition of basal diets (Qiu et al., 2021). In this study, it was important to highlight that the body weight of finishing pigs significantly increased after 14 days of 1% CHC supplementation compared to the control, although no significant difference was observed in carcass characteristics between the two groups. This was in agreement with previous studies demonstrating that CHC as a feed additive contributes to increased body weight gain during a short period, whereas its underlying mechanism is still unclear.

Intestinal microbes play an important role in regulating the growth characteristics and health status of the host such as fat deposition traits (Lei et al., 2021), chronic diseases (Coleman et al., 2018; Just et al., 2018), and disease resistance (Kumar et al., 2018). Increasing evidence suggests a functional and crucial role of nutrients from dairy foods in modulating the gut microbiota (Yang et al., 2017; Wang X. et al., 2019). In a study involving gilts, the administration of choline for 96 days resulted in a significant increase in the α-diversity of the gut microbiome, as evidenced by higher Chao1, Shannon, and Simpson indexes (Zhan et al., 2023). Interestingly, we found that a 14-day dietary supplementation of CHC did not significantly improve the Chao1 and Shannon indexes of the intestinal microbiota in finishing pigs; this may be attributed to the shorter duration of the feeding period. In contrast, the PCoA and NMDS analyses suggested a significant alteration of gut microbiota structure in finishing pigs after dietary CHC supplementation, supporting an altered relative abundance of bacteria at different taxonomic levels in CHC groups. Firmicutes and Bacteroidota are the predominant phyla in most mammals and are also the two main bacteria phyla involved in fat accumulation and the metabolism of the host (Nuli et al., 2019). In particular, a significantly higher abundance ratio of Firmicutes to Bacteroidota is generally regarded as a marker signal of obesity and obesity-associated complications (Zheng et al., 2021). In finishing pigs, we detected a significant increase in the ratio of Firmicutes to Bacteroidota after a short-term supplementation of CHC, which may partly explain why CHC pigs exhibited significantly increased body weight and carcass weight compared to the control pigs. Eubacterium has been reported to be involved in various metabolic processes, including the fermentation of dietary fibers and the production of short-chain fatty acids such as butyrate (Mukherjee et al., 2020), and plays a functional role in maintaining intestinal health in both humans and pigs, especially in improving growth performance and feed conversion efficiency in pigs (Sheridan et al., 2020). In line with previous findings that choline supplementation contributes to enhancing Eubacterium in sows (Zhan et al., 2023), a significant increase in carcass weight and relative abundance of Eubacterium were shown in the feces of CHC pigs compared with control pigs. In addition, the top three core microorganisms at the family level, namely, Prevotellaceae, Ruminococcaceae, and Lactobacillaceae are closely related to lipid metabolism in pigs (Li Y. et al., 2020). Of note, Prevotella has been considered a core microorganism mediating fat deposition in pigs. Increasing relative abundance of Prevotella may contribute to promoting feed intake but negatively correlates with lean meat percentage in pigs (Yang et al., 2018; Chen et al., 2021). Here, a marked reduction in the relative abundance of f_Prevotellaceae and g_Prevotella was found in fecal samples of CHC pigs when compared with controls, which may provide new insight into the functional role of CHC in the regulation of lipid metabolism in finishing pigs.

It is well-known that the liver not only serves as a pivotal organ engaged in the metabolism of nutrients, drugs, hormones, and toxins (Lassiter et al., 2018; Song et al., 2020) but also is the primary site of synthesis for various liver-related substances. To investigate the effect of CHC on the liver of finishing pigs, we performed a non-targeted metabolomics approach to examine whether CHC supplementation can cause a marked difference in the metabolite composition of the liver in finishing pigs. As expected, we observed a significant alteration of metabolites in the liver between two groups, and 84 metabolites were remarkably altered by CHC supplementation, of which most were predominantly identified as sphingolipids, amino acids, peptides, derivatives, and phosphatidyl. Notably, sphingolipids presenting in cell membranes are also classified as complex lipids and play a crucial role in cellular signal transduction, membrane structure maintenance, and lipid metabolism (Sartorio et al., 2022), which is consistent with observations in CHC-altered gut microbiotas mediating the lipid metabolism of the host. Among decreased metabolites responding to CHC treatment, Carnitine-C5:1 is often used as a biomarker for metabolic disorders, such as elevated levels that may indicate defects in fatty acid metabolism (Calabrese et al., 2006; Wang Y. et al., 2019). In addition, NAD is involved in key metabolic processes such as glycolysis, the Krebs cycle (also known as the citric acid cycle or TCA cycle), and oxidative phosphorylation (Canto et al., 2015; Harutyunyan et al., 2021). Consistently, the KEGG pathway analysis revealed that upregulated metabolites were mainly enriched in African trypanosomiasis, primary bile acid biosynthesis, aldosterone synthesis and secretion, taurine and hypotaurine metabolism, as well as thiamine metabolism. Previously, taurine and hypotaurine have been demonstrated to play important roles in various biological processes, such as the maintenance of the integrity and function of cell membranes. Enhanced taurine and hypotaurine metabolism may contribute to promoting fat digestion and absorption, mediating neural transmission and regulation, and protecting against oxidative stress (Davison and Kaczmarek, 1971; Wen et al., 2019; Baliou et al., 2021). Considering the higher taurine and hypotaurine metabolism of the liver in CHC pigs than that of control pigs, our data may suggest that the inclusion of CHC may exert a positive influence on taurine levels by modulating both taurine metabolism and glutathione metabolism. In contrast, we found that reduced metabolites from CHC exposure were mainly involved in nicotine addiction, GABAergic synapse, histidine metabolism, aminoacyl-tRNA biosynthesis, and nicotinate and nicotinamide metabolism. In pigs, nicotinate (nicotinic acid) and nicotinamide metabolism plays important roles in various physiological processes and overall health, which might be associated with the metabolism of carbohydrates, fats, and proteins (Gumpenberger et al., 2021; Udumula et al., 2021). Increased nicotinate (nicotinic acid) and nicotinamide can significantly improve the reproductive performance and growth of pigs. Moreover, similar to previous findings of CHC in the regulation of intracellular levels of reactive oxygen species, increased nicotinate and nicotinamide, and taurine and hypotaurine contribute to the prevention of oxidative stress (Jong et al., 2012). In this study, however, we only observed a decreasing trend in TG levels and slightly increased T-AOC, GSH-PX, and SOD levels in the liver, which may be because the ability of CHC to protect against reactive oxygen species will be significantly limited under normal conditions. Finally, Pearson correlation analysis indicated that p_Bacteroidota, g_Muribaculaceae, and g_Megasphaera were strongly positively correlated with sphingolipid and phosphatidyl acid metabolites. This finding further revealed that the symbiotic relationship between bacteroidota and sphingolipid metabolites in the intestines of finishing pigs, following a brief period of CHC supplementation, synergistically contributed to the enhancement of both growth and carcass performance. However, further studies are needed to explain the underlying mechanism by which CHC regulates the composition of gut microbiota and metabolites, contributing to improving the growth and health of finishing pigs.

## 5. Conclusion

Our results revealed that short-term supplementation of CHC contributes to increased body weight gain and carcass weight in finishing pigs, which may be involved in the regulation of gut microbiota and alterations of liver metabolism. These findings provide novel perspectives into the capacity of choline-mediated gut microbiota/metabolites to improve growth performance, carcass characteristics, and health.

## Data availability statement

The datasets presented in this study can be found in online repositories. The names of the repository/repositories and accession number(s) can be found at: https://www.ncbi.nlm.nih.gov/, PRJNA1009785.

## Ethics statement

The animal study was approved by the Ethics Committee of Sichuan Agricultural University (Sichuan, China, approval number 2021302172). The study was conducted in accordance with the local legislation and institutional requirements.

## Author contributions

ZX: Conceptualization, Data curation, Writing—original draft, Methodology. JD: Conceptualization, Data curation, Formal analysis, Writing—original draft. MG: Software, Data curation, Writing—review and editing. CZ: Validation, Writing—review and editing. ML: Validation, Writing—review and editing. CL: Software, Writing—original draft. MW: Validation, Writing—original draft. LC: Resources, Writing—original draft. YZ: Visualization, Writing—review and editing. YW: Investigation, Visualization, Writing—review and editing. YJ: Resources, Writing—review and editing. WC: Supervision, Writing—original draft. KZ: Supervision, Writing—original draft. YL: Supervision, Writing—original draft. LZ: Funding acquisition, Project administration, Supervision, Writing—review and editing. LS: Funding acquisition, Project administration, Supervision, Writing—review and editing.
