# Supplementary material for: Short-term dietary choline supplementation alters the gut microbiota and liver metabolism of finishing pigs
Source: Front Microbiol. 2023 Sep 28;14:1266042. doi: 10.3389/fmicb.2023.1266042 (PMC10569418; doi:10.3389/fmicb.2023.1266042)
Supplement: Supplementary file 1 [file Data_Sheet_1.zip › Supplementary material/Supplementary Figure1.docx]

Short-term dietary choline supplementation alters the gut microbiota and liver metabolism of finishing pigs

**Zhongwei Xie^1,2,†^, Junhua Du^1,2,†^, Mailin Gan^1,2^, Chengpeng Zhou^1,2^, Menglin Li^1,2^, Chengming Liu^1,2^, Meng Wang^1,2^, Lei Chen^1,2^, Ye Zhao^1,2^, Yan Wang^1,2^, Yanzhi Jiang^2,3^, Wenqiang Cheng^4^, Kangping Zhu^5^, Yi Luo^5^, Li Zhu^1,2^, Linyuan Shen^1,2,*^**

^1^ Key Laboratory of Livestock and Poultry Multi-omics, Ministry of Agriculture and Rural Affairs, College of Animal and Technology, Sichuan Agricultural University, Chengdu 611130, China

^2^ State Key Laboratory of Swine and Poultry Breeding Industry, Sichuan Agricultural University, Chengdu 611130, China

^3^ College of Life Science, Sichuan Agricultural University, Chengdu 611130, China;

^4^ National Animal Husbandry Service, Beijing 100125, China

^5^ Sichuan Dekon Livestock Foodstuff Group, Shuangliu 610200, China

*** Correspondence:**

Linyuan Shen^1,2,*^

shenlinyuan@sicau.edu.cn

**† These authors contributed equally to this work.**

**Supporting information**

**Figures**


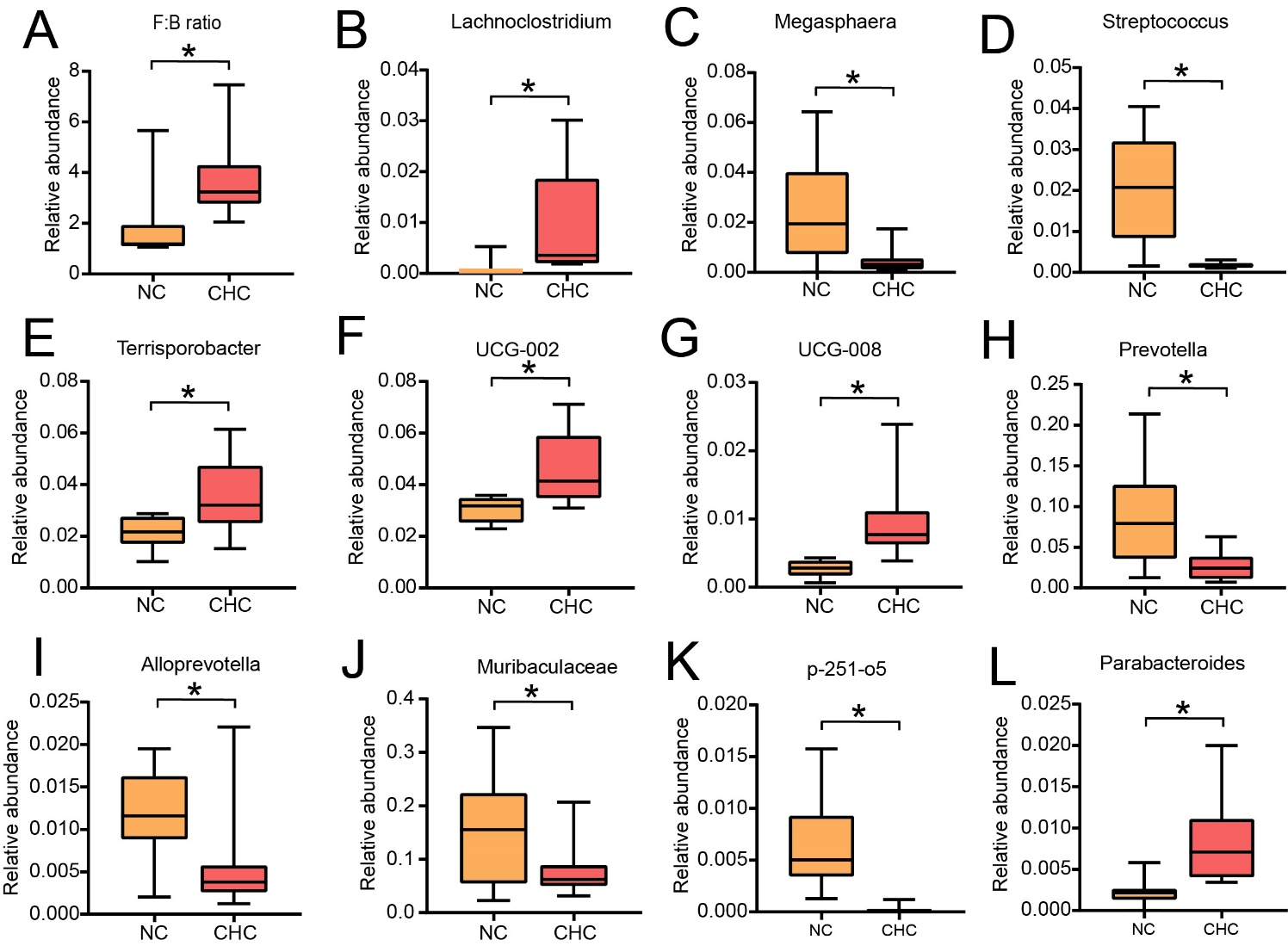


**Supplementary Figure 1.** The ratio of Firmicutes to Bacteroidota. (**A**) Significantly changes (p < 0.05) in the relative abundance of the Firmicutes/Bacteroidota at phylum taxa level. (**B-G**) Among the top 40 abundant genera, six of them belong to the Firmicutes phylum and showed significant changes. (**H-L**) Among the top 40 abundant genera, five of them belong to the Bacteroidota phylum and showed significant changes.
