# Supplementary material for: Short-term dietary choline supplementation alters the gut microbiota and liver metabolism of finishing pigs
Source: Front Microbiol. 2023 Sep 28;14:1266042. doi: 10.3389/fmicb.2023.1266042 (PMC10569418; doi:10.3389/fmicb.2023.1266042)
Supplement: Supplementary file 1 [file Data_Sheet_1.zip › Supplementary material/Supplementary Table1.docx]

Short-term dietary choline supplementation alters the gut microbiota and liver metabolism of finishing pigs

**Zhongwei Xie^1,2,†^, Junhua Du^1,2,†^, Mailin Gan^1,2^, Chengpeng Zhou^1,2^, Menglin Li^1,2^, Chengming Liu^1,2^, Meng Wang^1,2^, Lei Chen^1,2^, Ye Zhao^1,2^, Yan Wang^1,2^, Yanzhi Jiang^2,3^, Wenqiang Cheng^4^, Kangping Zhu^5^, Yi Luo^5^, Li Zhu^1,2^, Linyuan Shen^1,2,*^**

^1^ Key Laboratory of Livestock and Poultry Multi-omics, Ministry of Agriculture and Rural Affairs, College of Animal and Technology, Sichuan Agricultural University, Chengdu 611130, China

^2^ State Key Laboratory of Swine and Poultry Breeding Industry, Sichuan Agricultural University, Chengdu 611130, China

^3^ College of Life Science, Sichuan Agricultural University, Chengdu 611130, China;

^4^ National Animal Husbandry Service, Beijing 100125, China

^5^ Sichuan Dekon Livestock Foodstuff Group, Shuangliu 610200, China

*** Correspondence:**

Linyuan Shen^1,2,*^

shenlinyuan@sicau.edu.cn

**† These authors contributed equally to this work.**

**Supporting information**

**Supplementary Table 1**

The composition and nutrient levels of the basal diet.

| Feed ingredient | Nutrient levels | Content（%） |
| --- | --- | --- |
| Corn | Crude protein | ≥14.0 |
| Flour | Crude fiber | ≤8.0 |
| Wheat | Crude ash | ≤8.0 |
| Soybean meal | Calcium | 0.40-1.20 |
| Stone powder | Total phosphorus | ≥0.40 |
| Calcium hydrogen phosphate | Sodium chloride | 0.30-0.80 |
| Sodium chloride | Lysine | ≥0.65 |
| Vitamins and vitaminoids | Water | ≤13.0 |
| Mineral elements |  |  |
| L-lysine |  |  |
| Threonine |  |  |
| Mold inhibitor (calcium propionate) |  |  |
